# Men’s Social Connectedness in Later Life: A Qualitative Study with Older Men

**DOI:** 10.3390/geriatrics9020053

**Published:** 2024-04-19

**Authors:** Henrique Pereira, Patricia Silva, Renata Della Torre, Marta Rosário dos Santos, Adriana Moutinho, Sofia Solinho, Constança Proença, Joana Cabral, Ana Jorge Santos

**Affiliations:** 1 Department of Psychology and Education, Faculty of Social and Human Sciences, University of Beira Interior, Pólo IV, 6200-209 Covilhã, Portugal; pg.silva@ubi.pt (P.S.); renata.torre@ubi.pt (R.D.T.); marta.r.santos@ubi.pt (M.R.d.S.); adriana.s.g.moutinho4@gmail.com (A.M.); sofia.solinhodepeniche@gmail.com (S.S.); constanca.proenca.poco@ubi.pt (C.P.); anajorge@cmail.pt (A.J.S.); 2 Research Centre in Sports Sciences, Health Sciences and Human Development (CIDESD), 5001-801 Vila Real, Portugal; 3 Instituto Piaget, 3515-776 Viseu, Portugal; joanatavarescabral@gmail.com

**Keywords:** social connectedness, older men, later life

## Abstract

This qualitative study aimed to understand men’s social connectedness in later life in Portugal focusing on their perceptions, obstacles, strategies, and impact on well-being. The sample included 104 older Portuguese men over 65 years of age (*M_age_* = 70.76 years). The qualitative data were the direct transcriptions of the answers given by participants to the electronic interview using thematic analysis. Findings revealed six overarching themes encompassing 18 subcategories: definitions of social connectedness (social support, community identity, mental health promotion, use of community structures), difficulties/obstacles in maintaining social connectedness (ageism, lack of initiative, physical limitations, psychological traits, resources), strategies/actions or resources to establish social connections (use of technology, use of community groups, leisure and sport activities, church/religion), negative impact of difficulties in establishing relevant social connections (mental health, physical health, relationships), positive actions from being socially connected (positive prescriptions to promote social connectedness), and concerns from being socially disconnected (health risks). These findings indicate that the lack of social connectedness creates social vulnerability in later life, and social support is needed to ensure safer aging among older men.

## 1. Introduction

Social connectedness, defined as the experience of belonging to a social relationship or network [1], is an essential component of overall health and well-being at any age and for all genders [2]. However, being socially connected depends on various factors, including lifelong socialization patterns that are often rooted in cultural roles and norms. In this regard, gender differences have been observed across the aging process concerning social connectedness, indicating that older women tend to have larger and more diverse social networks and greater social support compared to older men [3]. Conversely, older men often maintain intimate relationships with only a few individuals, identifying fewer people as important to them [4], spending more time alone, and engaging less in formal group activities [5], thereby implying significant implications for their overall health status [6].

Furthermore, cultural background is extremely important when analyzing community and social network connections in determining older adults’ risks of loneliness [7]. As people age, more cultural backgrounds are acquired, allowing individuals to live based on their choices and adapt to their habitat through new learning [8]. But the process of forming relationships operates differently depending on cultural contexts. As Dong and Simon [9] reported in their study, rural subjects revealed lower levels of social support measures and quality of life, and higher levels of psychosocial burden, depressive symptomatology, and feelings of loneliness. Furthermore, widowhood, chronic work stress, poor mental and physical health and function, small social networks, poor-quality relationships, and financial issues, work as risk factors for loneliness, while more engagement with community activities, longer duration of residence, education, and positive marital relationships work as protective factors against loneliness [10,11].

This sociocultural background encourages older men in western societies to present with lower levels of social connectedness, measured as a reduction in the size of a person’s social network [12], due to several factors such as: limited socialization over the life course, the lack of peripheral social relations, the death of family members and friends, and the accumulation of trauma from losses occurred earlier in the life course [13], which are usually aggravated by retirement, thus constituting a risk factor in terms of feelings of loneliness, social isolation and lack of purpose in life. This typically leads to physical and mental health problems, including depression, suicidal behavior, and anxiety symptoms [14], especially when social disconnectedness is linked to perceived social isolation. 

Several studies have demonstrated how social disconnectedness is a risk factor for morbidity and mortality among older men, including the presence of health problems such as excessive alcohol drinking [15,16], poor global cognitive function, memory, and executive function [17], and overall mental health impairment, intensified by the COVID-19 pandemic [18]. On the other hand, it is well demonstrated how being socially connected is a protective factor to effectively adapt to social environments and remain socially active in later life, especially when older members of society tend to be recognized as resourceful and respectful mediators when fostering relationships within their communities [19]. In fact, this seems to be more the case when barriers such as ethnic differences, poor/limited mobility and lack of outdoor activities are overcome [20]. 

The reason behind why older men suffer more from social disconnectedness still needs further clarification, but recent research suggests heterogeneous trajectories in age-related changes in social connectedness, which affects around 6% of older people, such as living alone or with family members, having lower education levels, presenting cognitive impairment, fair/poor self-rated health, instrumental limitations, and depression [21]. Given their traditional role as breadwinners in most societies, men tend to have fewer social interactions and report smaller social networks than women [22]. Also, transitioning from active life to retirement [23], facing ageism, exposure to age-based discriminatory practices and internalization of age-related stereotypes [24], feeling stigma associated with belonging to an older sexual minority community [25,26], and experiencing stigma associated with changes in physical functioning and social roles, may all further inhibit their social networking and increase their social disconnection.

To promote social connection in older men, several interventions have been explored, with emphasis especially based on technology, but results appear contradictory. While some older male participants demonstrated fears and concerns related to Internet use [27], others stated that, by using a specific application, for example, they were able to build relationships with other men, thus improving their social connection [28]. Factors that contributed to this improvement included setting specific goals in using the technology and achieving visible health benefits. Another study found that participants reported feeling more connected to friends, family and their community when learning to use new technologies, highlighting the role of education and ongoing support in barriers to the technological adoption process [29]. Still, many older men do not have access to technology and those who do may have to deal with the consequences of a lack of human interactions, considering that many may face cognitive limitations or socioeconomic disadvantages [30]. However, due to the rapid pace of current technological change, many of the insights provided by previous studies about implementation of interventions with technology while important, are outdated [31].

As observed, it is crucial to comprehend the issues related to social connection for older men, especially considering the rapid population aging and demographic changes occurring in many countries. In Portugal, for instance, where this study was conducted, the older population tripled in the past three decades, becoming the fastest aging country in the European Union with an annual aging rate of 3.6%, or 182 older people (aged 65 years and above) for every 100 young people (aged up to 14 years) [32]. Also, most of the older population in the country are women, since over the age of 85, women represent 66.4% of the total population. This phenomenon can be explained by the greater longevity of women, who have a higher life expectancy at birth (83.4 years) than men (77.7 years) and, on the other hand, by the greater male mortality rate (12.6% in men versus 11.4% in women) in the country [33].

This distinct demographic profile in Portugal is also shaped by a wider process of socialization that tends to value social representations of hegemonic ideologies, creating invisibility in the mainstream media for older men in Portuguese society [34]. Hence, understanding men’s social connectedness in later life in Portugal requires clarification. Therefore, the objectives of this qualitative study were to find answers to the following research questions: (1) how do older men perceive social connectedness? (2) what obstacles/barriers prevent older men from establishing social connections? (3) what strategies, actions or resources do they use to establish and maintain social connections? and (4) what is the impact of social disconnectedness on their health and overall well-being?

## 2. Materials and Methods

### 2.1. Participants

In this qualitative study, we employed a purposive sampling strategy [35] to select a cohort comprising 104 older Portuguese men aged over 65. This approach was chosen based on Sandelowski’s proposition [36] that a qualitative research sample should facilitate a thorough analysis, leading to a nuanced and comprehensive understanding of the phenomenon under study, while avoiding excessive size or undue restriction. Consequently, the inclusion of 104 participants was determined through a formula that considered factors such as the study’s scope, participant diversity, methodology, and researcher expertise, as represented by the equation P = No-show rate × ((scope × diversity × method)/expertise). The inclusion criteria stipulated that participants must be aged 65 or more and have Portuguese as their mother tongue. All individuals meeting the inclusion criteria were considered eligible, since they could provide their informed consent and respond to the interview questions. However, given the online nature of our research, at least a small proficiency in technology use and sufficient internet access for participating in the electronic interview were essential.

### 2.2. Instruments and Procedures

Data collection was carried out between April and June 2023, through the dissemination of the survey on email lists, social networks and community organizations dedicated to older adults. Potential participants were contacted and invited to take part in the study, after they agreed to sign an informed consent agreement. Initially, a total of 980 people were contacted, but only 104 of them responded to the invitation to take part in the survey, resulting in a response rate of 10%. The online interviews were carried out in an asynchronous way, using a semi-structured questionnaire provided on the Microsoft Forms platform with an average completion time of 25 min. This type of research gives participants the flexibility to participate and facilitates the recruitment of participants, taking advantage of the opportunities offered by the Internet [37,38].

Participants were asked to complete a sociodemographic questionnaire, measuring questions such as age, education level, marital status, place of residence, or professional status. In addition, the questionnaire was composed of a set of five open-ended questions developed for the purpose of this study: (1) Social connections are very important throughout all stages of the life cycle, as they make it possible to break isolation and loneliness and, therefore, promote greater well-being among older adults. How does this apply to you, your life, and your circumstances as an older man? Please elaborate. (2) What difficulties/obstacles do you feel at this stage of your life, to establish relevant social contacts that make you feel good, important, and included? (3) What strategies/actions or resources do you use to establish social connections? For example, support groups, dating apps, community activities, etc.? Can you give examples? Please elaborate. (4) How do any difficulties in establishing relevant social connections at this stage of your life impact your life, your physical and mental health, and your general well-being? Please elaborate. (5) Would you like to add any ideas or personal experiences on this topic that you consider important for us? If yes, please elaborate.

All procedures were implemented in accordance with the Helsinki Declaration of 1975 and this study was approved by the Ethics Committee of the University of Beira Interior (Portugal). When accessing the platform, all participants were informed about the purpose of the study, the aspects related to confidentiality and anonymity and were provided with all the information necessary to take part in the study voluntarily. They were also informed that they could quit at any time, without any consequences. Finally, participants were also provided with the contact details of the research team in case they had any questions or concerns during the study. After this, the 104 respondents agreed to answer the questionnaire. All documents and data were coded, and participants were identified by fictitious names to protect their privacy. In addition, only the team responsible for data analysis had access to the responses of the participants. These precautions were implemented to ensure that the personal information of the participants remained protected and that their identity was not revealed at any stage of the study. 

### 2.3. Data Analysis

The transcription of the responses provided by the participants during the electronic interview was used to evaluate the qualitative data. Thematic analysis was employed to extract the semantic content and latent constructs in the narratives of our participants [39]. This process involved several steps, including: becoming familiar with the data through periodic reading and writing of preliminary concepts; creating initial codes to organize the relevant data; searching for themes by collecting similar and relevant data for each possible theme; reviewing themes that worked with the coded extracts; defining and naming themes through refining the specificities of each one; and producing the final report, including the final selection of vivid extracts [40].

The narrative responses were systematically analyzed by the authors, allowing for an analysis throughout a process of continuous comparison of recurring themes, considering the variety of disparity and degrees of each participant’s response. The consistency of coding was evaluated by comparing the codifications of two independent consultants, each with over 20 years of experience and necessary expertise. In cases of disparities in codifications, the two independent coders engaged in a discussion to reach a consensus. Translations of the original content of the electronic interviews conducted in the Portuguese language were carried out by a professional specialized in the field of health and aging, experienced and fluent in both English and Portuguese. Finally, the assessors defined and organized the broader final themes. To illustrate these themes, they also extracted portions of the interviews that represented these themes.

## 3. Results

### 3.1. Sociodemographic Characteristics 

The men had an average age of 70.76 years (*SD* = 5.45), ranging from 65 to 86 years. Table 1 describes in greater detail all sociodemographic information of the 104 participants in the study.

### 3.2. Thematic Analysis

The responses of the 104 participants revealed six overarching themes encompassing 18 subcategories. Table 2 describes each of the overarching themes, categories, and subcategories. Demonstrative quotes from the participants are provided.

#### 3.2.1. Definitions of Social Connectedness

Based on our participant’s definitions, social connectedness involved direct interactions with their social support, maintenance of a sense of community identity, maintenance of positive mental health status, and the use of community structures. The “Social Support” category includes maintaining family and friendship ties, as well as intergenerational contact, and the participants expressed the importance of maintaining close ties with family and friends of different age groups to cultivate a sense of utility, belonging and well-being. The “Community identity” addresses the importance of feeling part of a meaningful group and religious groups, and reveals how participation, especially in religious groups, provided spiritual comfort and meaningful social connections for some participants. The category of “Mental health promotion” emphasizes the role of social connection in preventing loneliness, social isolation, feelings of exclusion and feeling redundant. Finally, in the “Use of community structures”, some participants define social connectedness as the use of means such as social media and community organizations to strengthen interpersonal relationships. Overall, the definitions used provide an understanding of how our participants identify, value, and seek to maintain meaningful social connectedness in their lives.

*For me, being socially connected means maintaining daily connections with my family and friends, as this allows me to create a feeling of usefulness, belonging and well-being.*

(António, 72)

*I think it is essential to be attentive to new ideas from younger generations, even if they are not very open or available, but this means that we can maintain a certain level of update with new ideas, new ways of acting and emerging phenomena in society that let us to feel that we are connected.*

(João, 69)

*For me, being involved in my church is fundamental to feeling connected, as it is there that I meet my friends and obtain spiritual comfort.*

(José, 79)

*Being socially connected means not feeling alone or isolated. I think it’s very important that older people don’t feel redundant, and I do everything I can to avoid having that feeling of being excluded from society.*

(Pedro, 70)

#### 3.2.2. Difficulties/Obstacles in Maintaining Social Connectedness

Several difficulties and/or obstacles were mentioned by our participants in maintaining their social connectedness. Age-related prejudice emerges as a central theme, with many participants expressing the feeling of being discriminated against because of their advanced age, which directly influences their interest in engaging in social interactions. In addition, a lack of initiative is highlighted as a barrier, with participants mentioning a lack of time, availability, and energy to engage in social activities. In addition, physical limitations, including physical and sensory handicaps, and psychological challenges, such as temperament, low self-esteem, depression, and loneliness, have a direct impact on their willingness and ability to participate in social interactions, thus contributing to feelings of isolation and difficulties in establishing and maintaining meaningful connections. Finally, a lack of resources—financial, technological, and social—is also identified as a barrier to social connection, with some participants facing difficulties in accessing new technologies or seeing new technologies as a cause of dehumanization, or difficulty engaging in social activities due to financial restrictions or difficulties in finding people with similar interests.

*Society has become very prejudiced. Being older is seen as a burden, an expense to the government and to families, and being discriminated against daily due to your age directly affects my interest in becoming more involved in social interactions.*

(Paulo, 66)

*I feel like I don’t have the time or the availability to engage in social interactions.*

(Luís, 81)

*I have no interest in creating new social connections. It takes a lot of work to maintain the few ones that I presently have, and on the other hand, people discard you very easily based on today’s values in society.*

(Mário, 75)

*It worries me to say, but I honestly don’t have the energy to leave the house these days. It takes a lot of effort to present myself to the world, hygiene, dressing up, putting on a nice smile, and I rather stay at home, spend an afternoon reading a book or watching tv.*

(Alberto, 73)

*It’s not easy to admit, but aging is not without limitations. Health problems, hearing and eyesight problems, lack of stamina, it all interferes with the willingness to be with family members and friends. Sometimes they insist, but I say no to them just because I don’t want to impose my limitations on them.*

(Vítor, 81)

*The main reason why I don’t connect as much is my own self. I know I am not always easy to get along with, I tend to complain a lot when things don’t seem right, which is very often.*

(Hugo, 68)

*Having poor self-esteem and feeling lonely all the time contributed to my depression. It’s a new stage of my life where I feel demotivated, sad, and alone most of the time, and I simply stay away from people.*

(Jorge, 70)

*Having access to and knowing how to use new technologies is key to survive in today’s world. I use all kinds of social media and feel very connected through them. I interact with social friends from around the world and this helps me a lot with my being connected socially.*

(Rodrigo, 72)

*Being older and poor is one of the saddest things in the world. We reach a phase in our lives where you shouldn’t have to worry about money and material things, but this is not the case for me. My retirement is not sufficient to meet all my expenses, hence, I must avoid being socially connected to save up some money necessary to pay rent or medication.*

(Francisco, 70)

#### 3.2.3. Strategies/Actions or Resources to Establish Social Connections

Participants employ several strategies and use different resources to establish and maintain social connectedness as they get older. Most men described, for example, the use of technologies such as internet and social networks (Facebook, Instagram and TikTok), which they believe facilitate communication and interaction with others. In addition, the use of community groups, including support groups and professional organizations, was mentioned in order to connect with individuals who share similar interests, thus facilitating meaningful connections and social networks. In addition, engaging in leisure and sports activities was highlighted to promote social interaction and create connections. Participants mention activities such as going to restaurants, cafés, dance clubs, spas, attending cultural events or playing sports as opportunities to socialize and meet new people. Participation in religious events and activities is emphasized as a means of promoting social connections and finding a sense of belonging within a religious community.

*Instagram, Facebook, Tik-Tok, all are good excuses for me to feel connected. Every day, I use it to update information, keep up with the news of friends and relatives, comment on the interactions and feel alive, even if it all happens virtually.*

(Carlos, 70)

*I used to be an engineer and a professor, and I still maintain contact with former colleagues and students, as well as professional organizations in the field. This helps me feel useful, important, and good about myself, since I can still be of value to professionally active friends and colleagues.*

(Daniel, 78)

*I am old school, and I still get together with friends the old fashion way: I call them and ask: “do you want to come over? Do you want to go to the movies tonight? Do you want to have lunch? It’s easy and simple.*

(Manuel, 65)

*I try to be physically active; I visit the local gym 2 or 3 times a week, and I try to take different classes that help me stay physically and emotionally fit. At the same time, I get to see people there, interact with them, and share similar interests. This creates a nice feeling of belonging to a place.*

(André, 71)

*Church has always played an important role in my life, not only spiritually, but also, socially. Now, at this stage of my life, it is even more so, because at church I know that everybody cares about me. I feel cherished. Hence, I never miss an event, I contribute, and I hardly feel lonely.*

(Ivo, 80)

#### 3.2.4. Negative Impact of Difficulties in Establishing Relevant Social Connections

The negative impact of difficulties in establishing relevant social connections is evident among the participants, affecting various aspects of their lives. Mental health is particularly affected, with many experiencing feelings of depression, loneliness, disappointment, and a sense of being replaced by younger and more “vibrant” individuals. Some men express a profound lack of interest in life and a sense of resignation, feeling isolated and disconnected from society. Additionally, physical health is affected, and participants mentioned factors such as tiredness and the direct and indirect effects of the COVID-19 pandemic, which also played a significant role in aggravation of these difficulties. Finally, participants express the impact of social disconnection in their most intimate relationships, including a lack of sexual activities and difficulties in establishing or maintaining healthy relationships, leading to frustration and a desire for meaningful connections.

*Aging is not easy, especially when society makes you feel unworthy. It’s a feeling of being replaced by newer, more robust, more important people. I feel disappointed and sad about this.*

(Gil, 80)

*What can you do? We must accept the fact that nobody cares about us anymore. I don’t know if this is how things are meant to be, but I often feel alone in a desert island, sad, with no interest in life, waiting for my final breath.*

(Rui, 79)

*Well, I still have needs… I would still love to find someone to be intimate with. Finding a girlfriend at my age is a very hard thing, it looks nearly impossible. It’s frustrating because I would love to have a significant relationship.*

(António, 72)

#### 3.2.5. Positive Actions from Being Socially Connected

The participants recognize the need to be socially connected as beneficial and feel eager to recommend several actions that are positively associated with the promotion of social connectedness. Many men mention the importance of staying active and participating in different social and community networks, which allows them to expand their horizons and continue learning throughout their lives. There is also a need to value meaningful and relevant aspects, including creating/maintaining relevant intellectual activities in order to keep the mind stimulated, contributing to active ageing. In addition, working on maintaining pre-existing connections and keeping in touch with younger generations are considered important actions for promoting a sense of belonging and transmitting knowledge and experience to the next generations. The participants also consider it important to keep a relaxed attitude towards social connection, not to worry too much about what they can do or what they can say to maintain a flexible approach and a light responsibility towards social relationships. Many of the ideas revealed by the participants include the ability to adapt to changes in this phase of life, improving their resilience, maintaining relevant activities, and cultivating a positive attitude towards life as essential actions for facing the challenges of ageing and remaining socially connected.

*If there’s one thing that helps me through the aging process is that I am wiser and I can choose what is important from what is not. This helps me focus, be resilient, and have a good laugh at what life has in store for me.*

(Júlio, 68)

*I need to be active, be in touch with nature, and always boost my body and my brain with positive energy. I feel no need to dwell on past regrets and burdens.*

(Nuno, 74)

*Being in contact with younger generations is key. I enjoy being around their joie the vivre, learn from them, and teach them using the skills I accumulated from my own experiences.*

(Afonso, 70)

*I find it hard to understand why some people my age simply gives up on themselves. It is our responsibility to make connections, call friends and family, make new friends, and still be part of society.*

(Daniel, 78)

#### 3.2.6. Concerns of Being Socially Disconnected

Finally, several participants shared their concerns regarding being socially disconnected, especially in terms of health risks. Cognitive decline is a common concern, with many recognizing that social disconnection can contribute to cognitive decline such as Alzheimer’s disease. In addition, loneliness and isolation are seen as significant risk factors for mental health, with some participants expressing concern about the increase in depression due to a lack of social interaction. The excessive use of social media is also raised as a concern, with some participants describing a feeling of dehumanization when they spend too much time online, distancing themselves from meaningful face-to-face interactions. In addition, the separation between work and family life is seen as a source of dissonance in the very nature of social relationships. Changes in the roles of providers and socially active individuals challenge the perception of active participation in the community, raising additional concerns about the ability to maintain meaningful and satisfying social connections after these roles change.

*I know that being socially isolated will only lead to more isolation, depression, and Alzheimer’s disease. It’s like calling death.*

(Martim, 73)

*Sometimes I feel a bit dehumanized, because being on social media is not real life. Feels like keeping up appearances.*

(Mateus, 74)

*I think we are all trying to figure out what to do with this new circumstance in life: we are no longer socially active, but still want to be.*

(Manuel, 65)

## 4. Discussion

This study aimed to understand the dynamics of social connectedness in older men, highlighting the experiences, strategies and challenges associated with this process. Our results showed that different themes (N = 6) and sub-themes (N = 18) emerged in the participants’ narratives, revealing the complexities and impacts underlying the building and maintenance of social connections in old age. Understanding these results is important, as social connectedness is an important factor in human development [41] and can directly impact people’s quality of life [42,43], which is especially relevant in the case of older men, who are susceptible to experiencing greater loneliness and social isolation, which results in poorer physical and mental health and quality of life [44]. By examining these topics, we can gain insights into how to promote a more solid social connectedness adapted to the needs of older men that favors their quality of life in the long term. In this sense, the most significant innovation and contribution of this study resides in the adoption of an approach that relates different factors of social connectedness in older men, including impacts, challenges, and concerns, coping strategies and actions. In addition, the qualitative nature of the study allows for a more detailed analysis of the participants’ narratives, providing a better understanding of the complexities involved in individual experiences. This multifaceted approach offers new and fresh insights and a unique perspective regarding social connectedness among older men, expanding existing knowledge in this area, especially in the Portuguese context, where research on this topic is limited.

### 4.1. Biopsychosocial Health Impacts

Social connectedness has been consistently associated with several positive impacts on the biopsychosocial health of older men [19,32,35]. In our study, the participants also attributed positive meanings to social connectedness. For them, social connectedness was related to social bonding (family, community, intergeneration and through social media) and the sense of social belonging involved being part of a group. In fact, a strong and supportive social network can provide increased emotional support, promoting feelings of belonging, security, and value, as well as promoting opportunities for enriching social and intellectual activities [44]. Our participants also define social connectedness as a protective factor for mental health, preventing loneliness, social isolation, exclusion, and perceived worthlessness. These perceptions corroborate previous research that associates participation in social activities and involvement in community groups with a decrease in symptoms of depression and anxiety hopelessness and cognitive decline among the elderly [41,45,46], as well as contributing to a better self-perception of well-being and successful ageing [42,47,48] and in perceived life expectancy [35]. Finally, participants demonstrated an awareness of the positive impact of social connectedness on overall physical health which has already been pointed out in previous studies [41] and in studies with the same target audience [13,35]. This understanding is in line with the theory that biopsychosocial health is integrative and connected between social, physical, and mental well-being. It is also important to mention that although our participants did not address it directly, the quality of interpersonal relationships has been associated with a variety of positive outcomes, including reducing the risk of disease and promoting healthy behaviors [49,50,51].

On the other hand, a lack of social connectedness can have adverse consequences for the biopsychosocial health of older men. These problems are expressed through our participants’ reports of symptoms of depression, anxiety, negative feelings, isolation, fatigue, and relational difficulties, as well as concerns about the long-term mental and physical health risks of social disconnection. Studies have associated social isolation and loneliness with an increased risk of developing depression, anxiety, cognitive decline, and other mental health conditions in older people [44,45,48]. Furthermore, older men who experience social isolation can face additional difficulties, such as a sense of disconnection from the world around them and a decreased quality of life [44,45,49]. Physical health problems have also been closely related to social relationships [28,49,52]. For physical health, this is especially relevant due the fact that social isolation and chronic loneliness can lead to a more sedentary and less healthy lifestyle, exacerbating the risk of physical health problems such as cardiovascular disease, immune compromise, diabetes, and premature mortality [52,53]. 

### 4.2. Challenges Associated with Social Connection in Older Men

Older men face several specific challenges when it comes to building and maintaining social connections. One of these challenges is the social stigma associated with ageing, which can lead to the exclusion and marginalization of older people [54,55,56,57]. In fact, feelings of prejudice for being older are an obstacle to maintaining social connection for our participants. Ageism is considered a global challenge and a barrier to active ageing and can negatively affect older people’s social interactions and limit their opportunities to participate in meaningful social activities [54,55,56,57]. In addition, fear of social judgment or fear of appearing vulnerable when expressing their social needs can lead older people, especially men, to avoid seeking support and building new connections [54,55,56,57]. Another challenge faced by our sample is a lack of initiative or motivation to seek out new social connections. These challenges have already been identified in previous studies in which, for example, there was a decline in interest in social interactions due to life changes, past experiences of social rejection, retirement, or even feelings of discouragement in the face of perceived difficulties in making new friends [12,13,19,58,59]. In addition, changes in social structure, such as the loss of loved ones and friends, can decrease social interest for older men, increasing their risk of social isolation [52].

Physical and psychological limitations were also pointed out as obstacles to social connection for our participants. In fact, physical and mental health problems, highlighting chronic health conditions, mobility difficulties and mood disorders, can negatively impact older people’s ability to engage in social activities, making it even more difficult to build new social connections [20,60]. In addition, it is essential to consider the cultural and gender issues that can influence the challenges faced by older men in finding social connections. Traditional masculinity norms can limit emotional expression and the search for social support among men, making them less likely to seek help or share their concerns with others [61,62]. Similarly, cultural factors, such as social expectations and family relationship systems, can influence the way older men perceive and seek social connections [63]. There are also men who coincide with the typical gender role that society assigns them and others who do not, such as older men from the LGBTIQA+ community, which is also highlighted as a difficulty of socialization in older age since these people tend to be more marginalized [25,64]. 

Finally, the lack of financial, technological, and social resources also seems to influence the maintenance of participants’ social connection. In terms of financial issues, lower income, financial contributions, and retirement have already been associated with limiting older men’s ability to participate in social activities that require additional spending, such as traveling, eating out or participating in social clubs [65,66]. This also affects their ability to continue contributing to household expenses, which is related to a greater sense of belonging and socialization [67]. In terms of technology-related problems, although technological advances offer opportunities for social connectivity, they can represent a barrier for those who are unfamiliar with digital platforms or have limited access to the Internet [68,69]. In addition, excessive use of social media can lead to a sense of disconnection and isolation, especially when online interactions replace face-to-face contact [70]. These issues also were mentioned by our participants. Finally, in relation to social resources, changes in family structures and work patterns can influence opportunities for social interaction, especially for those who experience a decrease in community involvement after retirement [71]. 

### 4.3. Strategies and Prescriptions for Social Connectivity for Older Men

To overcome the challenges associated with social connectedness, older men of our sample employ a variety of strategies and approaches to be socially connected. For our participants, the use of technology including the internet and social media seems to be a facilitator of social connectedness. Leaving aside the challenges that have already been presented here, social media has been highlighted as a means for older people to maintain and expand their social networks. Technology offers older people the opportunity to connect with friends, family, and virtual communities, thus mitigating the effects of social isolation and loneliness [72]. In addition, online groups and discussion forums provide a safe space to share experiences, interests and seek emotional support among peers [73]. The use of dating apps and social networking sites allows older people to widen their social circle and meet new people with similar interests, providing an opportunity to connect [74,75]. This can be especially valuable for those who live in rural areas or have mobility difficulties and reflects the increasing integration of technology into the daily lives of older people and their willingness to explore new forms of social interaction [70,76].

Another strategy for social connection in our sample is participation in leisure, sporting, and intellectual activities. In previous studies, older people who get involved in recreational activities, such as sports, book clubs, art groups and cultural events, point to these activities as a way of expanding their social networks and meeting people with similar interests [48,77,78]. In addition, participation in different networks such as religious groups continue to be a strong strategy for older men in our sample. Churches, synagogues, mosques, and other places of worship serve as important centers of connection for older people [79,80]. These groups offer different activities and services, including volunteering, study groups and social events, which promote social interaction and mutual support among participants [81]. Similarly, contact with professional organizations, such as retirement associations or specific interest groups, allows older people to maintain social connections related to their past careers or current interests [82,83], as also mentioned by our sample. In addition to these strategies, it is important to recognize the role of family, community, and intergenerational relationships in the social connectedness of older men that participate in this study. Family and social ties, including relationships with spouses, children and grandchildren, friends and neighbors play a key role in promoting well-being, independence, and social cohesion among older people [48,78,80]. 

Finally, for our participants, the ability to adapt to change and cultivate a positive attitude towards life are considered essential strategies for maintaining social connectedness in old age. This includes valuing what is relevant in their lives, maintaining a flexible approach and light responsibility towards social interactions, consisting in a way of balancing the seriousness and commitment necessary for healthy and respectful social interactions with the easy-going nature that makes interpersonal relationships more pleasant and harmonious. Additionally, it includes being resilient in the face of adversity, promoting adaptability to the physical and mental changes that come with ageing, cultivating self-esteem and inner harmony, practicing meditation techniques, and being connected with nature. These strategies not only strengthen existing social bonds, but also help older men to face life’s challenges in a more positive and constructive way, contributing to more successful ageing. Previous studies have highlighted the importance of resilience, positive adaptation to change and cultivating an optimistic attitude to promote emotional and social adjustment in old age [33,80,84]. Furthermore, valuing the meaning and relevance of social interactions is in line with the theory of socioemotional selectivity, which posits that older people prioritize meaningful and supportive relationships to maximize emotional well-being [58,85,86]. The practice of meditation and connecting with nature have also been associated with mental and emotional health benefits in older adults and beyond, providing stress relief, greater subjective well-being, quality of life and a greater sense of connection with the environment [87,88,89].

### 4.4. Conclusions and Implications

This study explored the dynamics of social connectedness in older men, highlighting the challenges, strategies and impacts associated with this process, revealing the relevance of social connectedness for promoting the health and biopsychosocial well-being of these people. Participants shared a variety of factors that influence their ability to establish social connections. Physical and psychological health problems such as mobility difficulties and mood disorders were identified as significant barriers to social interaction, along with depressive symptoms, anxiety and worries about the future. These results highlight the importance of interventions that address both aspects in order to improve quality of life. The older men in our sample also face social challenges when trying to maintain meaningful relationships. These problems include the stigma associated with ageing, a lack of initiative or motivation to seek out new relationships, physical and psychological limitations, cultural and gender issues and a lack of financial, technological and social resources. 

On the other hand, social relationships were associated with several positive outcomes in the lives of the participants. These include providing emotional support and a sense of belonging to a group, preventing loneliness and social isolation, improving well-being and quality of life and reducing the risk of physical and mental health problems. In addition, older people who can adapt to change and develop a positive attitude towards life are more likely to maintain satisfactory social relationships. Older men use a variety of strategies to overcome difficulties in social relationships. These include using technology to communicate virtually with others, engaging in recreational, athletic and intellectual activities, participating in religious and community groups, communicating with professional organizations and valuing family, community and intergenerational relationships. In addition, the promotion of resilience, adaptation to change and positive attitudes towards life are considered important strategies for promoting satisfactory social relationships in old age. These findings provide important contributions to the literature on social connectedness in old age, broadening the understanding of this phenomenon. By recognizing and addressing these aspects, we can work to create more inclusive and supportive social environments for older men, promoting their well-being and quality of life. 

Furthermore, it is intriguing to examine the beliefs individuals acquire through the convergence of cultural transmission, intergenerational influences, and personal life experiences. These beliefs, whether concerning the appropriateness of certain actions at an older age or the need for socialization, are closely intertwined with the behaviors exhibited by older individuals, influencing their level of adventurousness, extroversion, and social engagement. By recognizing and supporting the diversity of experiences and preferences among older men, we empower them to make informed decisions regarding their health and well-being according to their individual needs. Moreover, it is crucial to understand that the impacts of social disconnection extend beyond the individual older men, with broader implications for society as a whole. Social isolation among the elderly can strain healthcare and social assistance systems, escalating the costs associated with caring for older individuals with mental and physical health needs. Consequently, addressing these challenges not only benefits older men but also contributes to a healthier society. This is particularly pertinent in the context of Portugal, which has one of the oldest populations in Europe due to increased life expectancy and declining fertility rates. The relevance of these findings extends not only to gerontology but also to other areas of study, health policies, and social practices aimed at promoting active and healthy aging.

### 4.5. Limitations and Future Directions

Although this study offers important insights, it is essential to recognize its limitations. The sample, although representative for a qualitative study (n = 104), is made up mostly of Portuguese men who are white/European, heterosexual, have children, live in urban areas, and are married, which limits the diversity of perspectives and experiences from other communities. This includes people of different races and ethnicities, who are often the target of negative experiences such as racism, discrimination, and violence throughout their lives, as well as older men from the LGBTQIA+ community, who tend to experience stigmatization of identities, loss of social status, social dislocation and decreased autonomy, physical strength, and mental resilience [25,77,90]. Furthermore, due to the qualitative nature of the research, the results reflect the perceptions of the participants and are subject to social desirability bias, where some participants may downplay socially undesirable attitudes, events, ideas and behaviors and over-report more desirable attributes to the results [86]. It is also important to consider that older men may face difficulties in expressing their feelings deeply due to structural sexism [63]. Moreover, as no exclusion criteria or elucidation regarding technology usage capability, sufficient internet access, examination of cognitive deficits, updated digital literacy, and household information were imposed, the limitations and gaps could inform the development of future studies on the topic to understand important and specific issues surrounding the elderly population. Finally, the qualitative approach adopted for data analysis may not fully capture the complexity of individual experiences, and some nuances may have been missed during thematic analysis. Despite these limitations, the study provides a deeper and richer understanding of the experiences and relational connections of older men and contributes to filling a gap in the literature on the subject.

Future research could benefit from a more diverse sample, including men from different ethnic and racial backgrounds, as well as those who live in rural areas or have different sexual orientations. This would allow for a more comprehensive analysis of the experiences and challenges faced by older men in diverse social and cultural contexts. In addition, longitudinal studies would make it possible to assess how changes in older men’s social networks over time affect their physical and mental health, providing insights into ageing and social connections. Furthermore, it would be important to further explore the intersection between social connectedness and other determinants of health, such as socioeconomic conditions, access to health services and social support. This could be accomplished through different methodological approaches, such as mixed methods, quantitative studies, or participatory methods, to ensure a broader and more inclusive understanding of the experiences of social connectedness in older men. Experimental research could include the findings of this study to develop interventions aimed at improving the social connectivity and quality of life of older men, considering their needs and preferences, and examining the impact of specific technological interventions, such as the impact of apps or programs on promoting men’s social relationships. This also includes exploring how technology and social media can be used effectively, especially by those who may face cognitive or socioeconomic limitations. In addition, it is important to study how older men’s social networks change as they age and to analyze the social support groups created specifically for them, as well as their potential impacts. 

## Figures and Tables

**Table 1:** Sociodemographic characteristics (*M_age_* = 70.76; *SD* = 5.45).

		n	%
Marital Status	Married	77	74.0
	Single	6	5.8
	Separated/Divorced	11	10.6
	Widower	10	9.6
Professional status	Retired	67	64.4
	Professionally active	37	39.6
Place of residence	Urban	94	90.4
	Rural	10	9.6
Children	Yes	87	83.7
	No	17	16.3
Socioeconomic status	Low	37	35.6
	Medium	49	47.1
	High	18	17.3
Religious	Yes	51	49.0
	No	53	51.0
Race/ethnicity	White European	97	93.3
	Other	7	6.7
Sexual orientation	Straight	90	86.5
	Bisexual	4	3.8
	Gay	10	9.7

**Table 2:** Overarching themes, categories, and sub-categories.

Overarching Themes	Categories	Sub-Categories
Definitions of Social Connectedness	Social Support	Maintenance of family/friends’ ties
		Intergenerational contacts
	Community identity	Feelings of belonging to a group of important people
		Religious groups
	Mental health promotion	Loneliness prevention
		Social isolation prevention
		Exclusion prevention
		Redundancy prevention
	Use of community structures	Using social media
		Using community organizations
Difficulties/obstacles in maintaining social connectedness	Ageism	Feelings of prejudice from being older
	Lack of initiative	Lack of time/availability to social interactions
		Lack of interest in creating new social connections
		Lack of energy to leave the house
	Physical limitations	Physical and sensory handicaps
	Psychological traits	Own temperament
		Low self-esteem
		Depression
		Loneliness
	Resources	Difficulties in accessing new technologies
		Lack of financial resources
		Difficulties in finding people with similar interests
		Use of social networks causes dehumanization
Strategies/actions or resources to establish social connections	Use of technology	Use of the internet/social networks
	Use of community groups	Use of support groups
		Contact with professional organizations Social arrangements
	Leisure and sport activities	Restaurants, cafes, dance clubs, spas, dancing events, parties, yoga, theatre, traveling, gym, etc.
	Church/religion	Participation in Religious events
Negative impact of difficulties in establishing relevant social connections	Mental health	Depression
		Loneliness
		Disappointment/feelings of being replaced
		Resignation
		Lack of interest in life
		Isolation
	Physical health	Tiredness
		COVID-19
	Relationships	Lack of sexual activities
		Difficulties in establishing/maintaining healthy relationships
Positive actions from being socially connected	Positive prescriptions to promote social connectedness	To be active and/or join different networks
		Connectedness with nature
		Meditations
		To value what is relevant
		To keep relevant intellectual activities
		To work on maintaining pre-existing connections
		Maintain few responsibilities, be carefree/easygoing about relationship
		Cultivate self-esteem/inner harmony
		To promote adaptability to physical and mental changes
		To be resilient
		To keep contacts with younger generations
Concerns from being socially disconnected	Health risks	Cognitive decline
		Loneliness
		Isolation
		Excessive use of social media
		Depression
		Separation of work/family life creates dissonance

## Data Availability

Data are available upon request.
